# LncRNA CBR3-AS1 regulates of breast cancer drug sensitivity as a competing endogenous RNA through the JNK1/MEK4-mediated MAPK signal pathway

**DOI:** 10.1186/s13046-021-01844-7

**Published:** 2021-01-25

**Authors:** Ming Zhang, Yan Wang, Longyang Jiang, Xinyue Song, Ang Zheng, Hua Gao, Minjie Wei, Lin Zhao

**Affiliations:** 1 Department of Pharmacology, School of Pharmacy, China Medical University, Shenyang, 110122 Liaoning Province China; 2 Liaoning Key Laboratory of molecular targeted anti-tumor drug development and evaluation; Liaoning Cancer immune peptide drug Engineering Technology Research Center; Key Laboratory of Precision Diagnosis and Treatment of Gastrointestinal Tumors, Ministry of Education; China Medical University, Shenyang, Liaoning Province China

**Keywords:** Chemoresistance, Breast cancer, CBR3-AS1, miR-25-3p, MAPK signaling pathway

## Abstract

**Background:**

Adriamycin (ADR) resistance is one of the main obstacles to improving the clinical prognosis of breast cancer patients. Long noncoding RNAs (lncRNAs) can regulate cell behavior, but the role of these RNAs in the anti-ADR activity of breast cancer remains unclear. Here, we aim to investigate the imbalance of a particular long noncoding RNA, lncRNA CBR3 antisense RNA 1 (CBR3-AS1), and its role in ADR resistance.

**Methods:**

Microarray analysis of ADR-resistant breast cancer cells was performed to identify CBR3-AS1. CCK-8 and colony formation assays were used to detect the sensitivity of breast cancer cells to ADR. Dual-luciferase reporter, RNA pulldown, IHC and western blot analyses were used to verify the relationship between the expression of CBR3-AS1, miRNA and target genes. For in vivo experiments, the effect of CBR3-AS1 on breast cancer resistance was observed in a xenograft tumor model. The role of CBR3-AS1 in influencing ADR sensitivity was verified by clinical breast cancer specimens from the TCGA, CCLE, and GDSC databases.

**Results:**

We found that CBR3-AS1 expression was significantly increased in breast cancer tissues and was closely correlated with poor prognosis. CBR3-AS1 overexpression promoted ADR resistance in breast cancer cells in vitro and in vivo. Mechanistically, we identified that CBR3-AS1 functioned as a competitive endogenous RNA by sponging miR-25-3p. MEK4 and JNK1 of the MAPK pathway were determined to be direct downstream proteins of the CBR3-AS1/miR-25-3p axis in breast cancer cells.

**Conclusions:**

In summary, our findings demonstrate that CBR3-AS1 plays a critical role in the chemotherapy resistance of breast cancer by mediating the miR-25-3p and MEK4/JNK1 regulatory axes. The potential of CBR3-AS1 as a targetable oncogene and therapeutic biomarker of breast cancer was identified.

**Supplementary Information:**

The online version contains supplementary material available at 10.1186/s13046-021-01844-7.

## Background

Worldwide, breast cancer is the most common malignant tumor in women [1–3]. Every year, an estimated 1 million new breast cancer cases are diagnosed worldwide. Adriamycin (ADR) is considered to be one of the most effective drugs for breast cancer, especially after tamoxifen treatment fails. However, its efficacy as a chemotherapeutic drug is greatly reduced due to intrinsic and acquired drug resistance during treatment [4, 5]. The underlying mechanisms resulting in ADR resistance in breast cancer remain poorly understood.

LncRNAs are a group of noncoding RNA molecules more than 200 nucleotides in length [6]. According to reports, lncRNAs are involved in many biological processes related to carcinogenesis and drug resistance [7–10]. For example, TINCR promotes epithelial-mesenchymal transition and trastuzumab resistance by targeting miR-125b [11]. HOTAIR mediates tamoxifen resistance by promoting estrogen receptor transcriptional activity [12]. DSCAM-AS1 mediates tumor progression and tamoxifen resistance by regulating the estrogen receptor (ER) [13]. However, the role of lncRNAs in the process of ADR resistance in breast cancer is still not fully explored.

LncRNA CBR3-AS1 is located in the antisense region of carbonyl reductase 3 (CBR3). It was initially discovered that CBR3-AS1 was abnormally expressed in prostate cancer cell lines and tissues [14–16]. Further studies showed that up-regulation of CBR3-AS1 expression promotes the proliferation of prostate cancer cells and inhibit apoptosis. Subsequent studies found that overexpression of CBR3-AS1 was observed in gastric carcinoma, esophageal squamous cell carcinoma, osteosarcoma, colorectal cancer and retinoblastoma [17–21]. However, the biological function and clinical significance of CBR3-AS1 in breast cancer drug resistance remain unclear.

In our study, we screened differentially expressed lncRNAs between ADR-resistant breast cancer cells and their parent cells. We found that the expression of CBR3-AS1 was up-regulated in ADR-resistant breast cancer cells, and its high expression was associated with poor prognosis of breast cancer. The overexpression of CBR3-AS1 promoted ADR resistance whereas knockdown of CBR3-AS1 expression significantly repressed it both in vivo and in vitro. Further studies showed that CBR3-AS1 functions as a ceRNA (competing endogenous RNA) by binding miR-25-3p. JNK1 and MEK4 are downstream proteins of the CBR3-AS1/miR-25-3p axis in breast cancer cells. Our results confirm for the first time that CBR3 promotes ADR resistance in breast cancer cells via regulation of the CBR3-AS1/miR-25-3p/MEK4/JNK1 axis, indicating that CBR3-AS1 has potential as a targetable oncogene and biomarker for breast cancer treatment.

## Methods

### Access and analysis of public data

GSE20685 was downloaded from the GEO dataset (http://www.ncbi.nlm.nih.gov/geo/). The whole genome expression profiles and clinicopathological information of human cancer were downloaded from The Cancer Genome Atlas (TCGA) (https://tcga-data.nci.nih.gov/). The expression of lncRNAs in the cell lines was obtained from the Cancer Cell Line Encyclopedia (CCLE) database (http://www.broadinstitute.org/ccle). The IC_50_ values of drug in multiple cell lines were obtained from the Genomics of Drug Sensitivity in Cancer (GDSC) database. All transcripts were normalized by log_2_ transformation. The Diana lncbase (http://carolina.imis.athena-innovation.gr/diana_tools) was used to predict the binding of miRNAs and lncRNAs. The bioinformatics analysis tool miRdb (http://www.miRdb.org/) was used to predict the binding of miRNA and mRNA 3′UTR. The RNAhybrid website (https://bibiserv.cebitec.uni-bielefeld.de/rnahybrid/) was used to predict the binding sites of miRNA and lncRNA. The bioinformatics website lncLocator (http://www.csbio.sjtu.edu.cn/cgi-bin/lncLocator.py) was used to predict the subcellular localization of lncRNAs.

The correlation between genes was evaluated through the Pearson correlation test. The differences between breast cancer tumor and normal samples were evaluated by unpaired t-test. The log-rank test was used to test the difference in survival rate among different groups of patients.

### Patient specimens

Breast cancer specimens (*n* = 96) were obtained from hospitalized patients from November 2008 to June 2009. All patients were diagnosed with breast cancer at the First Affiliated Hospital of China Medical University. Before surgery, the patient did not receive chemotherapy or radiotherapy. This study was approved by the Ethics Committee of China Medical University (Approval number: AF-SOP- 07-1.1-01).

### Cell culture

MCF-7, T47D, MDA-MB-231 and HEK-293 T cell lines were purchased from the Institute of Biochemistry and Cell Biology at the Chinese Academy of Sciences (Shanghai, China), and MCF-7/ADR cells were purchased from the Zhen Shanghai and Shanghai Industrial Co., Ltd. (Shanghai, China). The cells were cultured in L-15 medium, DMEM or RPMI 1640 medium (HyClone, USA) and cultured in a humidified incubator at 37 °C. Except for cells cultured in L-15 medium, all cells were incubated in an environment containing 5% carbon dioxide.

### Microarray analysis

Microarray analysis was performed by Shanghai OE Biotech (China) with the microarray Agilent Human lncRNA 4*180 K (Design ID: 076500). The raw microarray data were submitted to the GEO database (accession number: GSE155478). The lncRNAs were identified by the Gencode database (https://www.gencodegenes.org). The *p*-value and fold change value of the t-test were used to screen differential lncRNAs. The screening criteria were up-regulated or down-regulated log_2_FC ≥ 2.0 and FDR ≤ 0.05.

### Gene set enrichment analysis

Data on CBR3-AS1 expression were obtained from breast cancer patients with TCGA. Taking the median CBR3-AS1 expression as the cut-off point, breast cancer cases were divided into two groups. The index of gene sequencing was set as “Pearson”, the curve of the top set was set to 150, and all other parameters were set at the default values.

### Cell transfection

The cells were seeded on a 6-well plate and incubated for 24 h. The cells reached 70–80% confluence. Lipofectamine 3000 (Thermo Fisher Scientific, MA, USA) transfection reagent (5 μl) was dissolved in 125 μl of serum-free medium. Two micrograms of DNA was diluted with 125 μl of serum-free medium to prepare the DNA (Hanheng, China) premix, and then 10 μl of p3000 reagent was added and mixed well. After incubation for 5 min, the DNA solution was added to the cells. After 4 h, the medium was replaced with fresh medium containing 10% serum. The final transfection concentration of miRNA mimics (RiboBio, Guangzhou, China) was 50 μM, and the final concentration of siRNA (RiboBio, China) transfection was 50 μM. In order not to decompose RNA, p3000 was not used in the transfection process, and the other steps were equivalent to DNA transfection.

In the animal experiments, the plasmid was stably transfected into cells as described above. Then, 1 mg/L puromycin was added to the culture for 1 month, and stably transfected cell lines were selected for the experiment.

### Western blotting

Cells were lysed on ice in RIPA buffer (Beyotime, China). The lysate was centrifuged at 15000×g at 4 °C for 15 min. SDS polyacrylamide gel electrophoresis was used to isolate proteins before they were transferred onto PVDF membranes. The membrane was blocked in 5% skim milk and incubated with the primary antibody in TBST at 4 °C overnight. Then, the cells were incubated with a horseradish peroxidase-labelled secondary antibody at a concentration of 1:10000 for 1 h. Finally, chemiluminescence was carried out using a Microchemi 4.2 system (DNR Bioimaging System, Israel). The details of all antibodies used are shown in Table S1.

### Luciferase assays

HEK-293 T cells were transfected with MEK4–3′UTR-WT, MEK4–3′UTR-MU, JNK1–3′UTR-WT and JNK1–3′UTR-MU plasmids. At the same time, the four groups of cells were transfected with either NC mimic or miR-25-3p mimic to form eight experimental groups. Finally, each group of cells was transfected with the Renilla luciferase plasmid. After 36 h, the cells were harvested for luciferase analysis using a dual luciferase reporting and detection system (Promega, USA). The results were normalized against Renilla luciferase activity.

### qRT-PCR

TRIzol (Invitrogen, USA) was used to extract total RNA from cultured cells. For mRNA and lncRNAs, a ReverTra Ace qPCR RT Kit (Toyobo, Japan) was used, and 200 ng of total RNA was used to synthesize cDNA in a 10 μl reaction volume. For miRNAs, the Bulge-Loop™ miRNA qRT-PCR Starter Kit (RiboBio, China) was used, and 1 μg of total RNA was used to synthesize cDNA in a 10 μl reaction volume. qRT-PCR of mRNA and lncRNAs was performed using SYBR Green Real-time PCR Master Mix (Toyobo, Japan) in a 12.5 μl reaction volume. The qRT-PCR of miRNAs used Bulge-LoopTM miRNA qRT-PCR Starter Kit (Ribobio, China) in a 20 μl reaction volume. The primers used are listed in Table S2.

### Colony formation assay

MCF-7/ADR and MCF-7 cells (1 × 10^3^/well) in the logarithmic growth phase were resuspended and seeded into a 60 mm cell culture dish. For the drug treatments, 100 nM ADR was added to MCF-7 cells and 30 μM ADR was added to MCF-7/ADR cells for 48 h. Afterward, the medium in the culture was replaced with normal medium. Two weeks later, the cells were washed with PBS twice, fixed with methanol, and stained with 0.1% crystal violet (Beyotime, China) for 30 min. The concentration of JNK1 inhibitor (SP600125, MedChemExpress, USA) used in the cell experiment was 40 nM.

### CCK8 assay

MCF-7/ADR and MCF-7 cells that were transiently transfected were seeded into 96-well plates at 3 × 10^3^ cells per well. After 24 h, the cells were treated with ADR (Sigma Chemical Co, St. Louis, MO) for 48 h. Next, cells were incubated with 10 μl of CCK-8 reagent (Dojindo, Japan) for 1 h before the absorbance was measured at 450 nm on a spectrophotometer.

### ADR accumulation assay

According to the method of our previous articles [22], a cumulative ADR measurement assay was performed. Briefly, the cells were exposed to 5 μM ADR for 2 h and then washed with PBS. Next, flow cytometry was used to measure ADR fluorescence to determine the intracellular ADR level.

### Flow cytometric analysis of apoptosis

MCF-7/ADR and MCF-7 cell apoptosis after ADR treatment was evaluated using the Annexin-V APC/7AAD Iodide Detection Kit (BD Biosciences, USA) according to the manufacturer’s instructions. Cells were then analysed by MACSQuant (Miltenyi Biotec, Germany).

### Animal assays

The negative control plasmid (GFP alone) or OE-CBR3-AS1 plasmid were transfected into MCF-7 cells, which were then treated with puromycin to obtain stably expressing cell lines. All BALB/c mice (4 weeks old) were purchased from Shanghai Laboratory Animal Centre (Shanghai, China). MCF-7/control and MCF-7/CBR3-AS1 cells (1 × 10^7^ cells/100 μl/nude mice) were subcutaneously injected into the axillary region of nude mice. When the subcutaneous tumors grew to 200–250 mm^3^ (16 days later), nude mice in the treatment group were intraperitoneally injected with ADR (2 mg/kg) and/or JNK1 inhibitor (15 mg/kg; MedChemExpress, USA) once every 3 days. All nude mice were treated 6 times. The diameter of the transplanted tumor was measured with digital calipers every week. The tumor volume was calculated as follows: volume = 0.5 × width^2^ × length. All nude mice were humanly sacrificed at the end of the experiment, and the tumor tissues were collected for further study by qRT-PCR, IHC and western blotting.

### ISH (in situ hybridization) and IHC (immunohistochemistry)

The assay was carried out according to the method mentioned in a previous study [23]. We collected breast cancer tissues from patients from the First Affiliated Hospital of China Medical University. The ISH probes were ordered from BOSTER Biological Technology Co., Ltd. (USA). For the IHC assays, we collected breast cancer tissues from different groups of subcutaneous. The details of all antibodies used are shown in Table S1.

### Biotin pull-down

MCF-7 cell lysates were incubated with CBR3-AS1 or control probe (Sangon, China) conjugated to streptavidin agarose resin beads (Thermo Fisher Scientific, USA) to generate probe-bound Dynabeads. After the samples were washed with buffer, DNase I (20 U) was added, and purified RNA was collected. The purified RNA was analysed by qRT-PCR. The whole experimental process and the buffer formulation were described by Hsu et al. [24].

### Statistical analysis

Quantitative data are expressed as the means ± SD of at least three independent experiments. All experimental values were evaluated using GraphPad Prism 8.3.0 (GraphPad software). The unpaired t-test was used for statistical analysis between the two experimental groups. The relationship between CBR3-AS1 expression and clinicopathological factors was tested by the chi square test. In all cases, *P* < 0.05 was considered statistically significant.

## Results

### CBR3-AS1 is up-regulated in drug-resistant breast cancer cells and is related to drug sensitivity and poor prognosis of breast cancer

**Fig. 1:**
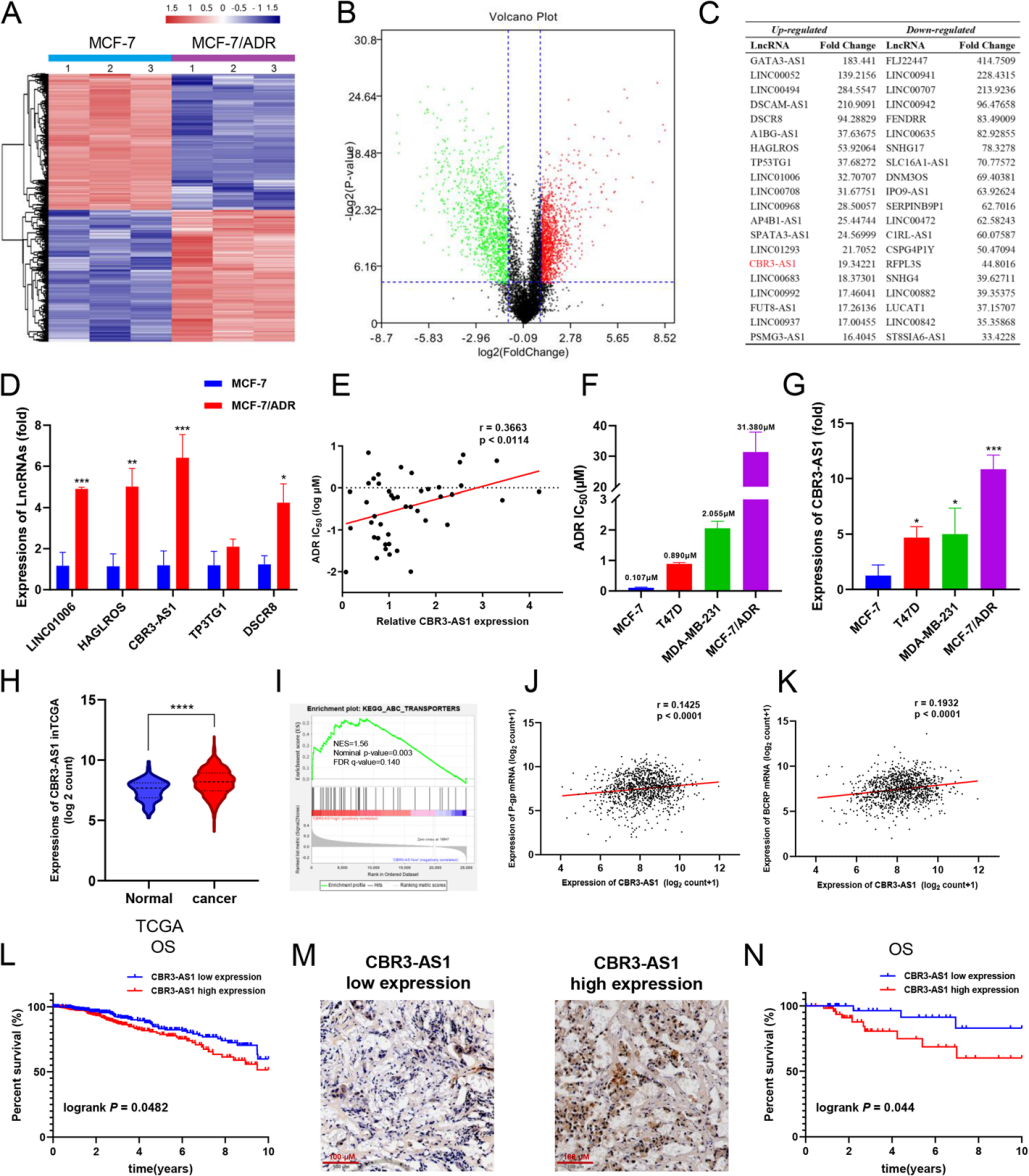
CBR3-AS1 expression is up-regulated in breast cancer-resistant cells and is related to drug sensitivity and poor prognosis of breast cancer. **a**, **b** Gene chip analysis of lncRNA transcripts in MCF-7/ADR cells and MCF-7 cells. **c** The top 20 lncRNAs with the most obvious up-regulation and downregulation in MCF-7/ADR cells. **d** qRT-PCR analysis of the expression of five lncRNAs in MCF-7/ADR cells and MCF-7 cells. **e** The correlation between CBR3-AS1 expression and the IC_50_ value of ADR in 44 breast cancer cell lines in the CCLE and GDSC databases. **f** The IC_50_ value of ADR was detected for both sensitive and resistant cells by the CCK-8 assay. **g** CBR3-AS1 expression in four breast cancer cell lines. **h** Expression of CBR3-AS1 in normal tissues and breast cancer tissues from the TCGA database. **i** GSEA analysis of CBR3-AS1 enrichment in the ABC transporter signaling pathway. **j**, **k** Correlation analysis between CBR3-AS1 and both P-gp mRNA and BCRP mRNA from the TGCA database. **l** Kaplan-Meier survival analysis based on CBR3-AS1 expression in breast cancer patients (*p* = 0.0482). **m** CBR3-AS1 expression in breast cancer patients as determined by ISH. Original magnification, × 200. Scale bars, 100 μm. **n** Kaplan-Meier survival analysis of breast cancer patients based on CBR3-AS1 expression in our cohort (*n* = 96, *p* = 0.044). *p < 0.05, **p < 0.01, ***p < 0.001, ****p < 0.0001

To identify whether the differential expression of lncRNAs can be induced during the course of breast cancer drug resistance, we investigated the expression of lncRNAs in MCF-7 cells and drug-resistant MCF-7/ADR breast cancer cells. LncRNAs with log_2_FC > 1 and an FDR (false discovery rate) < 0.05 were selected from the microarray data. The results showed that, compared with MCF-7 cells, MCF-7/ADR cells had 949 up-regulated lncRNAs and 915 downregulated lncRNAs (Fig. 1a-b). The top 20 lncRNAs with the most obvious up-regulation and downregulation in MCF-7/ADR cells are listed in Fig. 1c. Moreover, in the three groups of MCF-7/ADR cells and MCF-7 cells, differential expression of lncRNAs was used for principal component analysis (PCA). The results showed that the differential expression of lncRNAs could effectively distinguish MCF-7/ADR and MCF-7 cells (Fig. S1 A). We further selected five lncRNAs (DSCR8, TP53TG1, CBR3-AS1, LINC01006, HAGLROS) that were not previously reported to be related to the drug sensitivity of breast cancer and performed qRT-PCR to verify their expression levels (Fig. 1d). Among them, CBR3-AS1 showed the highest expression in MCF-7/ADR cells. To confirm the relationship between the expression of the five lncRNAs and the sensitivity of breast cancer cells to ADR, we observed the expression patterns of lncRNAs in various breast cancer cells from the CCLE database and then obtained the IC_50_ values of ADR in these breast cancer cells from the GDSC database. The results showed that the expression of CBR3-AS1 was positively correlated with the resistance of breast cancer cells to ADR (*p* < 0.05, *r* > 0.3) (Fig. 1e, Fig. S1B). To verify this result, we performed CCK-8 experiments on four breast cancer cell lines. The IC_50_ value of ADR in each cell line was calculated (Fig. 1f). The qRT-PCR results showed that CBR3-AS1 was highly expressed in breast cancer cell lines with high IC_50_ values but showed reduced expression in cell lines with a low IC50 value (Fig. 1g). In addition, we obtained the mRNA expression profile and clinical information of breast cancer patients from the GSE20685 dataset in the GEO dataset and used the Kaplan-Meier plotter website to calculate whether the survival of patients with breast cancer with different expression patterns of the five lncRNAs was different. The results showed that in breast cancer patients receiving chemotherapy, the prognosis of patients with high expression of CBR3-AS1 was worse (Fig. S1C). We also found that in the TCGA database, CBR3-AS1 was up-regulated in breast cancer tissues compared with adjacent normal tissues (Fig. 1h). Gene set enrichment analysis (GSEA) showed that CBR3-AS1 was enriched in the ABC transporter signal transduction pathway (Fig. 1i). In addition, the expression of CBR3-AS1 was significantly correlated with the mRNA expression of p-glycoprotein (P-gp, ABCB1) and breast cancer resistance protein (BCRP, ABCG2) (Fig. 1j, k). The log-rank test of the OS curve of breast cancer patients in the TCGA also indicated that compared with low CBR3-AS1 expression, high CBR3-AS1 expression was significantly correlated with worse OS (Fig. 1l). We evaluated the expression of CBR3-AS1 via ISH in 96 breast cancer samples from the First Affiliated Hospital of China Medical University. High and low expression is shown in Fig. 1m. There was a significant correlation between CBR3-AS1 level and both tumor size and pathological staging (Table 1). The log-rank test of the overall survival curve of these breast cancer patients showed that elevated expression of CBR3-AS1 was significantly related to the worse OS of breast cancer (Fig. 1n).

**Table 1:** The relationship between the expression of CBR3-AS1 and the clinicopathological characteristics of BRCA patients

Factors	Number (96)	CBR3-AS1 expression		χ2	^a^*p*-value
		Low (42)	High (54)		
Age (years)					
< 50	44	20	24	0.0959	0.7568
≥ 50	52	22	30		
Tumor size (cm)					
< 3	46	26	20	5.854	0.0155*
≥ 3	50	16	34		
ER					
positive	49	20	29	0.35	0.5541
negative	47	22	25		
PR					
positive	50	19	31	0.9567	0.328
negative	46	23	23		
Her2					
positive	51	22	29	1.92E-05	0.9965
negative	44	19	25		
Molecular typing					
Luminal A	33	15	18	0.5994	0.8966
Luminal B	32	13	19		
Her-2	18	9	9		
Basal-like	13	5	8		
staging					
I	22	15	7	7.127	0.0283*
II	44	17	27		
III	30	10	20		

* *p* < 0.05

^a^Chi-square test

### Effect of CBR3-AS1 on the drug sensitivity of breast cancer cells

**Fig. 2:**
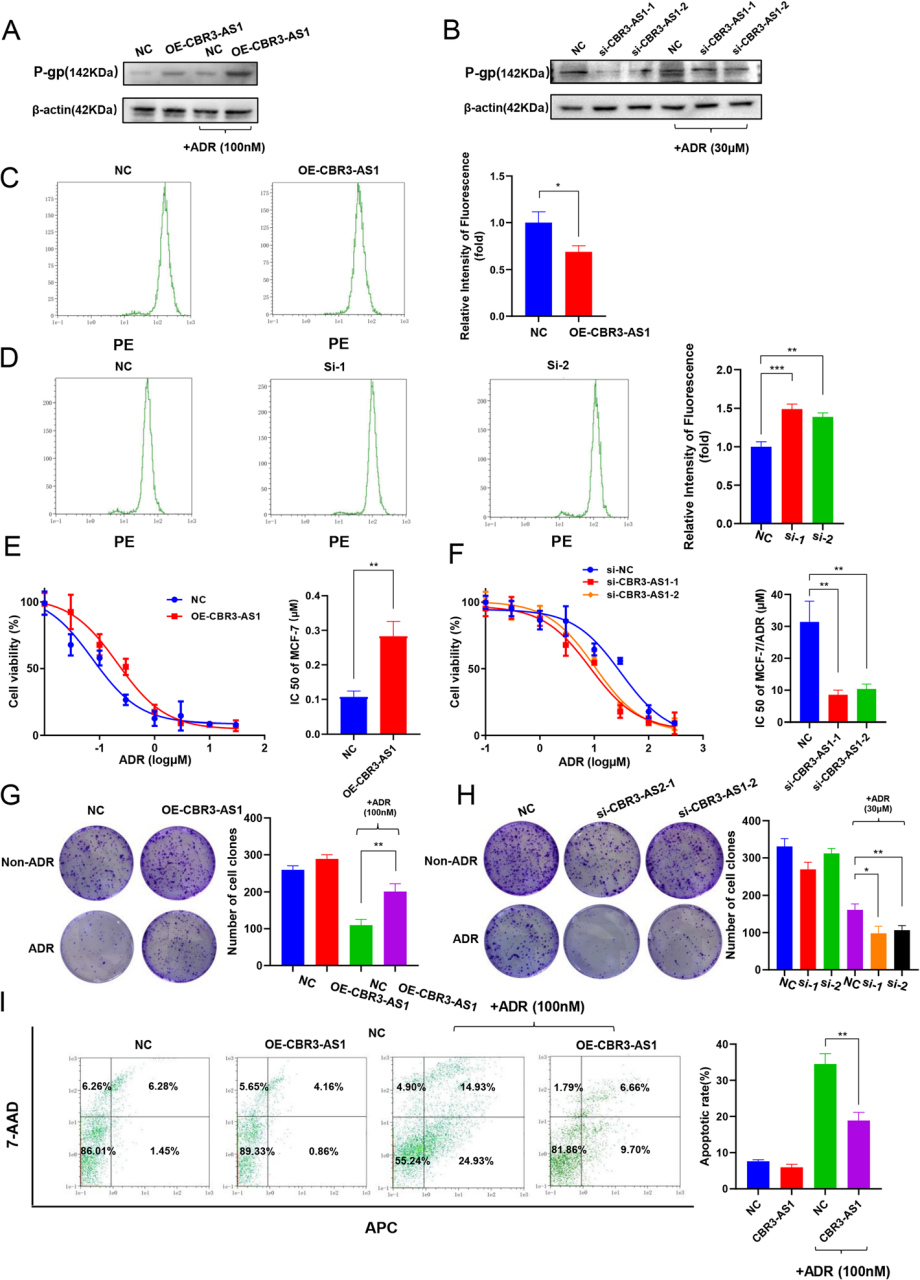
CBR3-AS1 affects breast cancer cell drug resistance. **a**, **b** P-gp expression was measured by western blot after CBR3-AS1 was overexpressed or silenced. The drug treatments comprised 100 nM ADR to MCF-7 cells and 30 μM ADR to MCF-7/ADR cells before culture for 48 h. **c**, **d** The fluorescence intensity of ADR was detected by flow cytometry, showing intracellular ADR accumulation. **e**, **f** Drug resistance was examined by CCK-8 assays in cells transfected with CBR3-AS1 overexpression plasmid or siRNAs. **g**, **h** Cells transfected with the CBR3-AS1 overexpression plasmid or siRNAs were seeded onto plates. The number of colonies was counted on the 14th day after seeding. **i** Apoptosis rates of MCF-7 cells treated with 100 nM ADR were detected. *p < 0.05, **p < 0.01, ***p < 0.001, ****p < 0.0001

To identify the effect of CBR3-AS1 on the drug sensitivity of breast cancer cells, we overexpressed CBR3-AS1 in MCF-7 cells with low expression of CBR3-AS1 and silenced CBR3-AS1 in MCF-7/ADR cells with high expression of CBR3-AS1. The overexpression or silencing efficiency of CBR3-AS1 was evaluated by qRT-PCR. After transfection of the CBR3-AS1 overexpression plasmid, CBR3-AS1 levels were significantly increased compared with those in the control group (Fig. S2A). Upon completion of si-CBR3-AS1 siRNA transfection, CBR3-AS1 expression was significantly downregulated compared with that in the si-control group (Fig. S2B). The results showed that in cells overexpressing CBR3-AS1, the protein expression levels of P-gp increased significantly (Fig. 2a). After CBR3-AS1 was silenced, the protein expression levels of P-gp decreased significantly (Fig. 2b). Then, we used flow cytometry to detect drug accumulation in cells. The overexpression of CBR3-AS1 significantly reduced the accumulation of ADR in cells (Fig. 2c), while silencing of CBR3-AS1 significantly increased the accumulation of ADR in cells (Fig. 2d). Next, we used the CCK-8 cytotoxicity test to detect changes in the drug sensitivity of cells. After overexpressing CBR3-AS1, cells exhibited a decrease in drug sensitivity (Fig. 2e, Table 2). However, after CBR3-AS1 was silenced, the cells’ drug sensitivity was significantly increased (Fig. 2f). We performed CCK-8 cytotoxicity experiments with other anticancer drugs in cells overexpressing CBR3-AS1. The results showed that after overexpressing CBR3-AS1, MCF-7 cells showed decreased sensitivity to paclitaxel decreased, while their sensitivity to cisplatin did not change (Fig. S2C, D). The above results showed that the effects of CBR3-AS1 on drug sensitivity are not specific to ADR. Colony formation experiments showed that colony-forming ability of cells was significantly enhanced by overexpression of CBR3-AS1 after adding ADR (Fig. 2g). However, compared with the control cells, si-CBR3-AS1 cells showed reduced colony-forming ability (Fig. 2h). At the same time, the IC50(48 h) concentration of ADR was used to induce apoptosis, and flow cytometry showed that CBR3-AS1 overexpression significantly reduced apoptosis (Fig. 2i).

**Table 2:** CCK8 assay on MCF-7 cells and MCF-7/ADR cells

medicine	IC_**50**_ (μM) ± SD				
	MCF-7	MCF-7/OE-CBR3-AS1	MCF-7/ADR	MCF-7/ADR-si-CBR3-AS1-1	MCF-7/ADR-si-CBR3-AS1-2
**ADR**	0.11±0.02	0.28±0.05	30.16±3.13	8.54±2.65	10.37±1.94

### CBR3-AS1 regulates MEK4 / JNK1 expression through miR-25-3p

**Fig. 3:**
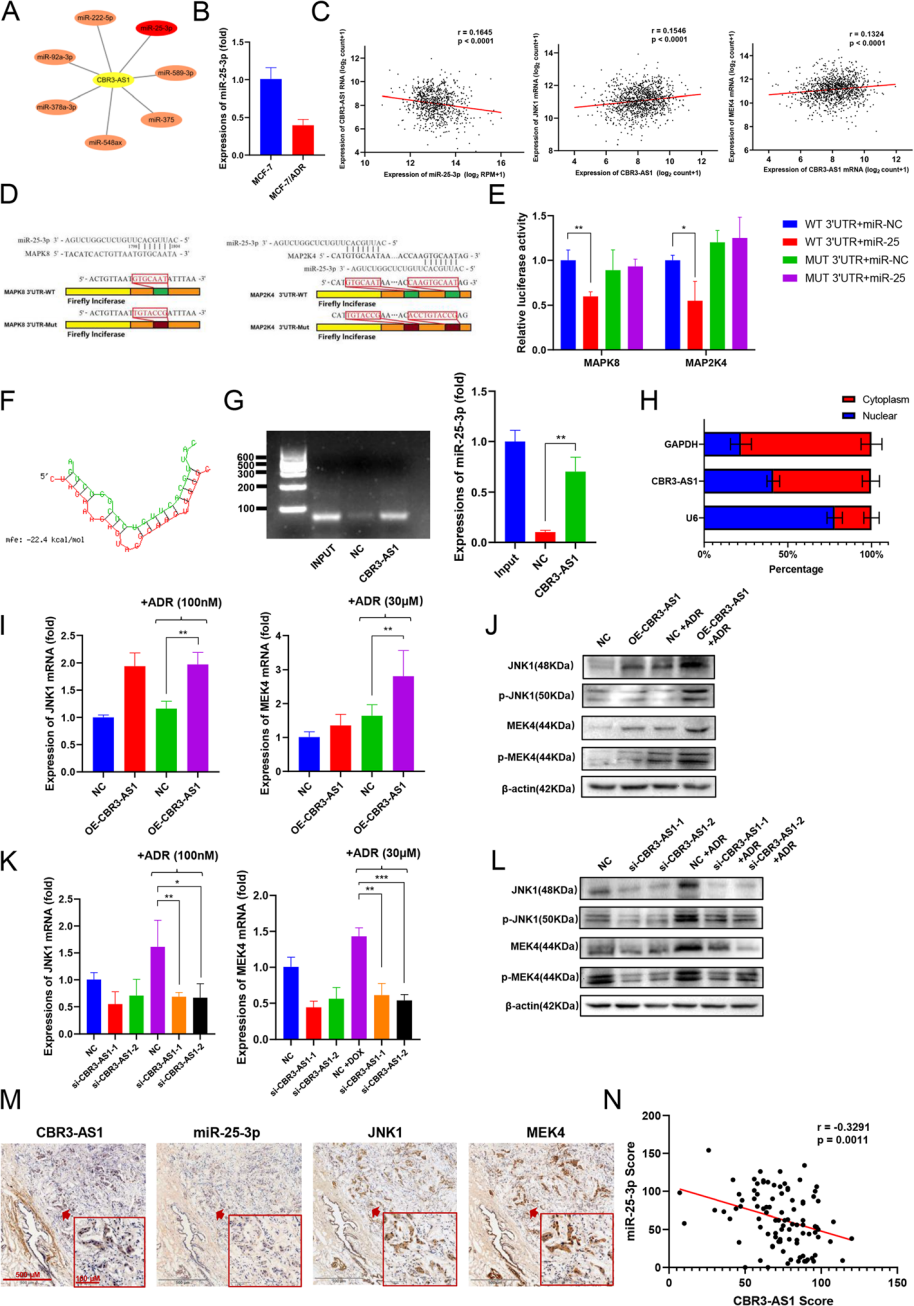
CBR3-AS1 functions as a sponge for miR-25-3p in the MAPK pathway. **a** DIANA-LncBase website showing CBR3-AS1-targeted miRNAs. **b** The expression of miR-25-3p in MCF-7 and MCF-7/ADR cells. **c** The correlation of CBR3-AS1 with miR-25-3p and JNK1/MEK4 mRNA expression in the TCGA breast cancer data. **d** The binding sites for miR-25-3p in the wild-type 3′UTR of JNK1 and MEK4 mRNA and the mutated sites in the 3′UTR of JNK1 and MEK4 mRNA. **e** Relative luciferase activity by luciferase reporter assays in MCF-7 cells co-transfected with wild-type (JNK1/MEK4-WT) or JNK1/MEK4-MUT and miR-25-3p or miR negative control (NC). **f**, **g** RNA pulldown shows that CBR3-AS1 directly binds with miR-25-3p. **h** RNA from the nucleus and cytoplasm of MCF-7 cells was extracted separately, and the proportion of CBR3-AS1 contained in the cell fractions was detected by qRT-PCR. **i**-**j** JNK1/MEK4 expression was measured by western blot and qRT-PCR after CBR3-AS1 was overexpressed in MCF-7 cells. **k-l** JNK1/MEK4 expression was measured by western blot and qRT-PCR after CBR3-AS1 was silenced in MCF-7/ADR cells. **m** ISH detected the expression of CBR3-AS1 and miR-25-3p, and IHC assays determined JNK1 and MEK4 expression in breast cancer patient tissues (*n* = 96). **n** Linear correlation pattern showing a negative relationship between the expression of CBR3-AS1 and miR-25-3p. *p < 0.05, **p < 0.01, ***p < 0.001, ****p < 0.0001

The bioinformatics analysis tool Diana lncbase revealed that lncRNA CBR3-AS1 targeted miRNAs (Fig. 3a, Fig. S3A). Research has shown that activation of miR-25-3p can overcome chemotherapy resistance [25]. qRT-PCR analysis showed that miR-25-3p expression was downregulated in MCF-7/ADR cells (Fig. 3b). The bioinformatics analysis tool miRdb was used to discover the 3′UTR binding site for miR-25-3p with JNK1/MEK4 in the MAPK pathway (Table S3). CBR3-AS1 showed a significant negative correlation with miR-25-3p and positive correlation with JNK1/MEK4 mRNA expression in the TCGA breast cancer data (Fig. 3c). To determine whether JNK1 and MEK4 are possible targets for miR-25-3p, a luciferase reporter gene assay was conducted. We constructed luciferase reporter vectors Luc-JNK1 and Luc-MEK4 containing the 3′UTRs of JNK1 and MEK4 (Fig. 3d). Overexpression of miR-25-3p significantly reduced the luc-jnk1 luciferase activity in MCF-7 cells (Fig. 3e), indicating that JNK1 and MEK4 are direct targets of miR-25-3p. The RNAhybrid website predicted that CBR3-AS1 has a binding site with miR-25-3p (Fig. 3f). RNA pulldown assays showed that CBR3-AS1 could directly bind to miR-25-3p in breast cancer cells (Fig. 3g). Since the function of lncRNAs depends on their subcellular distribution, we examined the subcellular localization of CBR3-AS1. The bioinformatics website lncLocator predicted that CBR3-AS1 was expressed in both the nucleus and cytoplasm (Fig. S3B). The results were verified by qRT-PCR (Fig. 3h). qRT-PCR analysis and western blot analysis showed that the overexpression of CBR3-AS1 increased the mRNA and protein expression of JNK1 and MEK4 in MCF-7 cells after ADR was added (Fig. 3i, j), while silencing CBR3-AS1 reduced the mRNA and protein expression of JNK1 and MEK4 in MCF-7/ADR cells (Fig. 3k, l). Moreover, overexpression of miR-25-3p decreased JNK1 and MEK4 expression in MCF-7/ADR cells (Fig. S3C, D), while silencing miR-25-3p expression may increase JNK1 and mek4 expression in MCF-7/ADR cells (Fig. S3E, F). We next used ISH and IHC to examine 96 breast cancer tissues, sliced into serial sections (Fig. 3m). Consistent with the results in breast cancer cells, a negative correlation was confirmed to exist between CBR3-AS1 and miR-25-3p within this cohort (Fig. 3n), the expression of CBR3-AS1 was positively correlated with JNK1/MEK4 (Fig. S3G) and miR-25-3p was negatively correlated with JNK1/MEK4 (Fig. S3H). In conclusion, CBR3-AS1 regulates the expression of MEK4/JNK1 through miR-25-3p.

### Silencing JNK1 or overexpressing miR-25-3p restored ADR drug sensitivity in breast cancer cells after overexpressing CBR3-AS1

**Fig. 4:**
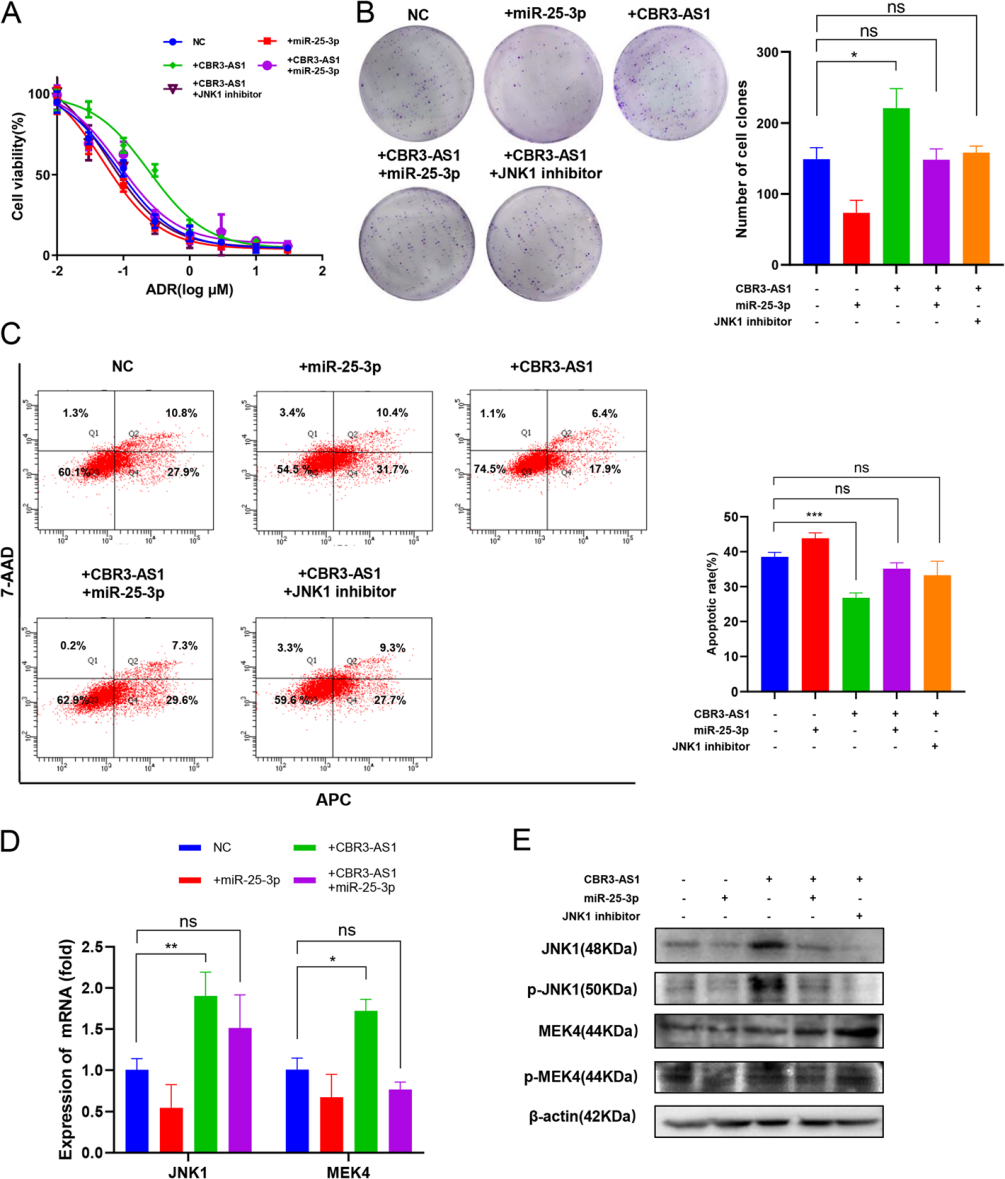
miR-25-3p overexpression reverses CBR3-AS1-induced activation of the MAPK pathway and chemotherapy resistance in breast cancer cells. **a** Drug resistance was examined by CCK-8 assays in MCF-7 cells transfected with the CBR3-AS1 overexpression plasmid, miR-25-3p mimic or JNK1 inhibitor. **b** MCF-7 cells transfected with the CBR3-AS1 overexpression plasmid, miR-25-3p mimic or JNK1 inhibitor were seeded onto plates. The number of colonies was counted on the 14th day after seeding. **c** Apoptosis rates were detected at a concentration of 200 nM ADR in MCF-7 cells when CBR3-AS1 and miR-25-3p were overexpressed or when JNK1 was inhibited. **d-e** JNK1, MEK4 and P-gp expression was measured by western blot and qRT-PCR in cells overexpressing CBR3-AS1 and miR-25-3p. *p < 0.05, **p < 0.01, ***p < 0.001, ****p < 0.0001

To determine whether miR-25-3p and JNK1 are involved in CBR3-AS1-mediated chemotherapy resistance in breast cancer cells, a CCK-8 assay was conducted with MCF-7 cells, and the results showed that overexpression of miR-25-3p or inhibition of JNK1 reversed the decrease in ADR sensitivity caused by overexpression of CBR3-AS1 (Fig. 4a). Colony-forming experiments confirmed that overexpression of miR-25-3p or inhibition of JNK1 reversed the increased cell proliferation by overexpression of CBR3-AS1 in cells treated with ADR (Fig. 4b). Apoptosis experiments confirmed that overexpression of CBR3-AS1 led to a decrease in the number of apoptotic cells, which was reversed by overexpressing miR-25-3p or inhibiting JNK1 (Fig. 4c). qRT-PCR analysis and western blot analysis showed that overexpression of miR-25-3p reversed the activation of JNK1 and MEK4 caused by overexpression of CBR3-AS1 (Fig. 4d-e).

### In vivo experiments showed that overexpression of CBR3-AS1 promoted the drug resistance of breast cancer cells to ADR

**Fig. 5:**
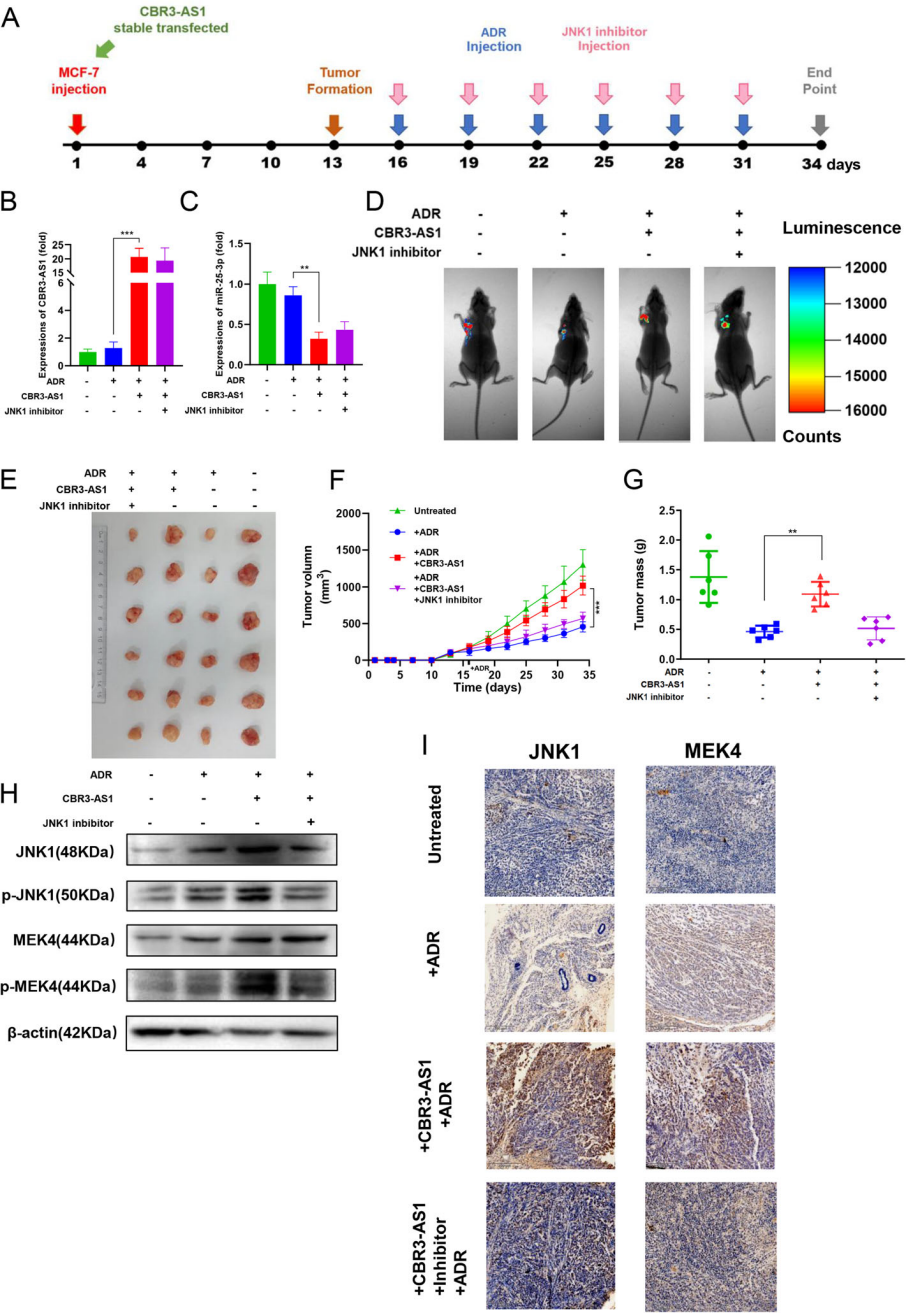
Overexpression of CBR3-AS1 promotes ADR resistance in vivo. **a** In vivo experimental design. **b** CBR3-AS1 expression in the transplanted tumors. **c** miR-25-3p expression in the transplanted tumors. **d** Live imaging experiments showing transplanted tumor cells. **e** Representative images of tumors at 5 weeks after subcutaneous transplantation when mice were euthanized. **f** Tumor growth curves of the respective groups. **g** Tumor mass of the respective groups. **h** JNK1 and MEK4 protein expression was measured in the respective groups by western blot. **I** IHC analysis of the expression levels of JNK1 and MEK4 in the respective groups. *p < 0.05, **p < 0.01, ***p < 0.001, ****p < 0.0001

To determine whether the overexpression of CBR3-AS1 can reduce the sensitivity of tumors to ADR in vivo and whether this effect is achieved by regulating JNK1, MCF-7 cells stably transfected with CBR3-AS1-cDNA or NC-cDNA were subcutaneously injected into nude mice. A schematic of the experiment is shown in Fig. 5a. After 34 days, the expression level of CBR3-AS1 in subcutaneous tumor tissue was detected. The expression level of CBR3-AS1 in the transfected group was still significantly higher than that in the control group, confirming the construction of the model (Fig. 5b). Next, the expression level of miR-25-3p in the tumor was detected, and it was found that the expression level of miR-25-3p in the OE-CBR3-AS1 group was significantly reduced (Fig. 5c). In vivo imaging in mice showed that overexpression of CBR3-AS1 resisted the inhibitory effect of ADR on tumor cells, which was reversed by inhibition of JNK1 (Fig. 5d). Furthermore, the effect of CBR3-AS1 on increasing tumor size and weight could be reversed by a JNK1 inhibitor (Fig. 5e-g). Western blot analysis showed that overexpression of CBR3-AS1 increased the expression of JNK1 and MEK4 (Fig. 5h). JNK1 and MEK4 expression was also detected by IHC (immunohistochemistry) (Fig. 5i). These results showed that CBR3-AS1 promotes ADR resistance in breast cancer through JNK1/MEK4 in vivo.

## Discussion

In the past few decades, breast cancer has been treated with standard chemotherapy after surgical resection, which greatly improves the overall survival of patients. However, cancer-related deaths are still on the rise due to breast cancer metastasis and drug resistance. The MAPK pathway plays a key role in the invasion, migration and metastasis of various malignant tumors [26–29]. Shen et al. found that overexpression of lncRNA PCAT-1 can enhance cell division and inhibit apoptosis in myeloma cells through the p38 and JNK MAPK pathways [30]. Xu et al. found that ROR2 facilitates epithelial-mesenchymal transition of breast cancer by regulating the MAPK/p38 signaling pathway [31]. Another study reported that circ-MAPK4 plays a key role in the survival and apoptosis of glioma cells by regulating the miR-125a-3p and p38/MAPK signaling pathways [32]. Another study reported that circ-MAPK4 plays a key role in the survival and apoptosis of glioma cells by regulating the miR-125a-3p and p38/MAPK signaling pathways [33].

JNK1 is one of the important genes in the MAPK pathway. MAPK pathway genes act as integration points of various biochemical signals and participate in many biological processes, such as differentiation, proliferation, apoptosis and transcriptional regulation [34, 35]. .MEK4 can phosphorylate the tyrosine residues of cytoplasmic JNK, resulting in its activation. Activated JNK quickly and significantly accumulates in the nucleus and causes the phosphorylation of the downstream transcription factors c-Jun, ATF-22, and Elk21; this in turn promotes the expression of related genes [36].

Some studies have shown that CBR3-AS1 is involved in and regulates the occurrence and development of cancers. It has been reported that CBR3-AS1 promotes osteosarcoma tumorigenesis and predicts poor prognosis [17]. CBR3-AS1 promotes the development of colorectal cancer by regulating the PI3K/Akt signaling pathway [18]. CBR3-AS1 plays an important role in the proliferation of esophageal squamous cells [19]. CBR3-AS1 is highly expressed in retinoblastoma and mediates the proliferation of retinoblastoma cells by regulating CBR3 [21]. However, it is not clear how CBR3-AS1 affects the drug resistance of breast cancer cells.

**Fig. 6:**
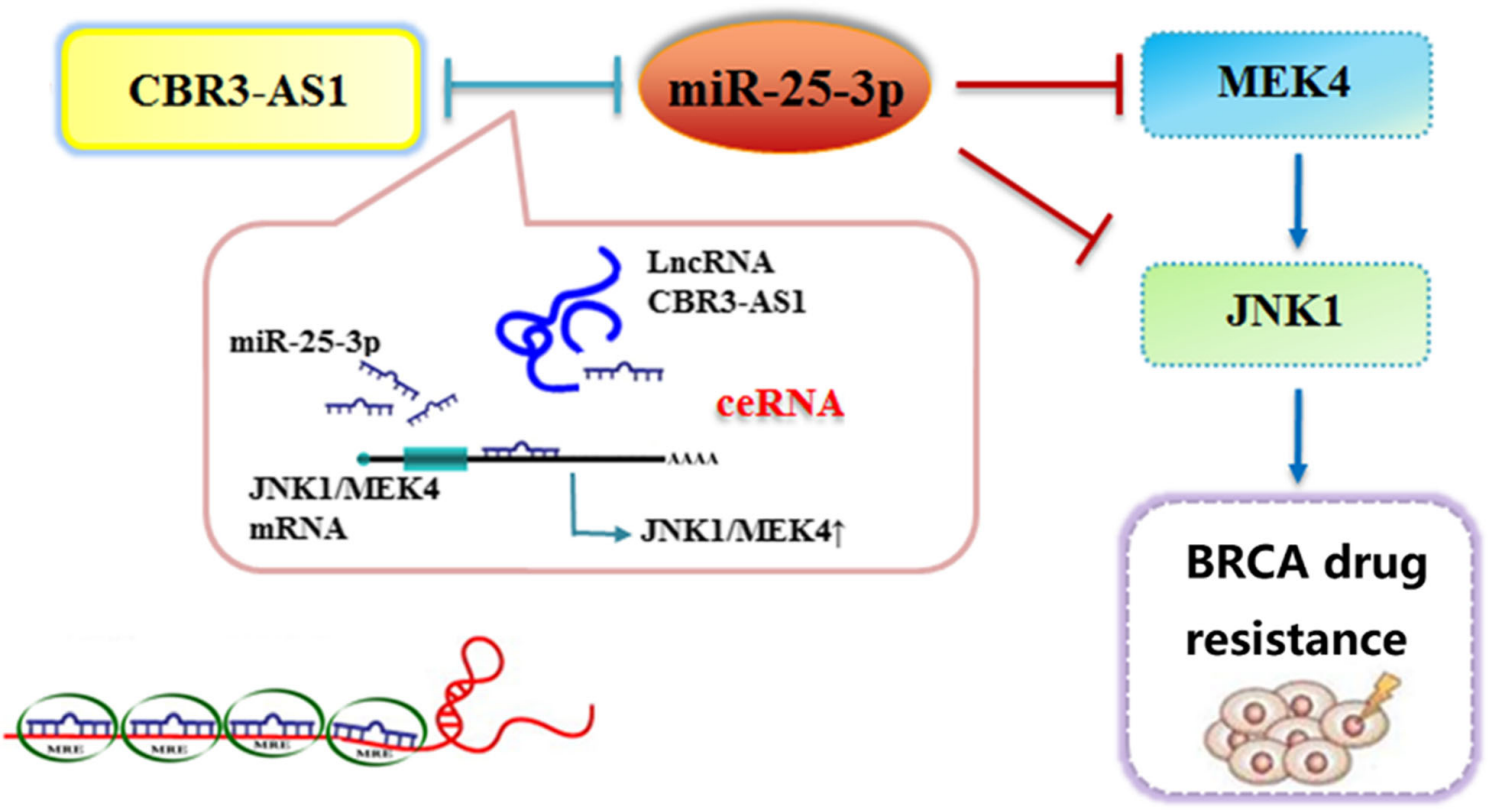
The schematic diagram indicating that CBR3-AS1 and JNK1 / MEK4 competitively bind to miR-25-3p, thus regulating MAPK signaling pathway to promote drug resistance of breast cancer cells

The ceRNA (competitive endogenous RNA) hypothesis suggests that a large-scale transcriptional regulatory network may be formed among RNAs, which can affect biological processes [23]. Our study is based on the ceRNA hypothesis, that is, cbr3-as1 directly binds to miR-25-3p, thus targeting JNK1/MEK4, activating the MAPK pathway, and ultimately affecting the development of chemoresistance in breast cancer (Fig. 6).

In this study, we determined that CBR3-AS1 plays a novel role in breast cancer cell resistance. Overexpression of CBR3-AS1 confers chemotherapy resistance to breast cancer cells in vitro and in vivo. It is worth noting that CBR3-AS1 expression is up-regulated in breast cancer, while patients with high levels of CBR3-AS1 show poor overall survival. These findings indicate that CBR3-AS1 can be used as a predictor of drug response in breast cancer patients and may be a useful biomarker for precision medicine in the clinic.

Our study has some limitations. We did not detect the effect of CBR-AS1 on MCF-7/ADR apoptosis. This is because to induce apoptosis in these cells apoptotic, a very large dose of ADR is needed. Because ADR itself has strong fluorescence, large amounts of ADR will interfere with commonly used fluorescent dyes such as FITC, 7-AAD, PE and APC, and it is difficult to interpret the results. Similarly, we did not use MCF-7/ADR in animal experiments. Due to the cardiotoxicity of ADR, it is difficult for mice to bear such a large dose. MCF-7/ADR cells also have difficulty forming tumors in mice.

## Conclusions

CBR3-AS1 might be an important biomarker for evaluating the prognosis of breast cancer patients. CBR3-AS1 increased the resistance of breast cancer to ADR by competitively binding miR-25-3p with JNK1/MEK4 and enhancing the MAPK signaling pathway. The CBR3-AS1/miR-25-3p/JNK1/MEK4 axis provides insight into breast cancer drug resistance mechanisms and theoretical support in the search for new breast cancer diagnostic markers and specific therapeutic targets.

## Supplementary Information

**Additional file 1: Figure S1.** CBR3-AS1 is related to breast cancer drug resistance and poor prognosis. A PCA analysis of the microarray of MCF-7/ADR cells and MCF-7/ADR cells. B The correlation between the expression of lncRNAs and the drug resistance of breast cancer cells to ADR. C Kaplan–Meier analysis were performed on patients of the expression of 5 lncRNAs in GSE20685.

**Additional file 2: Figure S2.** Construction of the cell models that interferes with the expression of CBR3-AS1. A After transfection of CBR3-AS1 plasmid, the expression of CBR3-AS1 in MCF-7 cells. B After transfection of CBR3-AS1 siRNAs, the expression of CBR3-AS1 in MCF-7/ADR cells. **C** The relative viability of the MCF-7 cells in cisplatin was detected by CCK-8 assays 48 h after transfection with Control and OE-CBR3-AS1. **D** The relative viability of the MCF-7 cells in paclitaxel was detected by CCK-8 assays 48 h after transfection with Control and OE-CBR3-AS1. *p < 0.05, **p < 0.01, ***p < 0.001, ****p < 0.0001.

**Additional file 3: Figure S3.** The relationship between CBR3-AS1, miR-25-3p and JNK1/MEK4. A Diana lncbase predicted the miRNAs combined with CBR3-AS1. **B** Subcellular localization of CBR3-AS1 predicted by the lnclocator website. C-D JNK1/MEK4 expression was measured by qRT-PCR after inhibited miR-25-3p in MCF-7 cells. E-F JNK1/MEK4 expression was measured by western blot and qRT-PCR after overexpressed miR-25-3p in MCF-7/ADR cells. G Linear correlation pattern showing a positive relationship between the expression of CBR3-AS1 and JNK1/MEK4. H Linear correlation pattern showing a negative relationship between the expression of miR-25-3p and JNK1/MEK4. *p < 0.05, **p < 0.01, ***p < 0.001, ****p < 0.0001.

**Additional file 4: Table S1.** The sequences for primers used in the study.

**Additional file 5: Table S2.** Antibodies used for IHC and WB.

**Additional file 6: Table S3.** The mRNAs banding to miR-25-3p.

## Data Availability

The datasets generated and analysed during the current study are available in the Gene Expression Omnibus data sets (https://www.ncbi.nlm.nih.gov/gds) (GSE155487).

## Abbreviations

TCGA: Cancer Genome Atlas
ADR: Adriamycin
CCK8: Cell Counting Kit-8
GEO: Gene Expression Omnibus
OS: Overall survival
FC: Fold change
MAPK: Mitogen-activated protein kinase
JNK: c-Jun N-terminal kinase
MEK: Mitogen-activated extracellular signal-regulated kinase
IHC: Immunohistochemistry
ISH: In situ hybridization
IC50: Half maximal inhibitory concentration
CBR3-AS1: Carbonyl reductase 3 antisense RNA 1
