# Supplementary figures and images for: LncRNA CBR3-AS1 regulates of breast cancer drug sensitivity as a competing endogenous RNA through the JNK1/MEK4-mediated MAPK signal pathway
Source: J Exp Clin Cancer Res. 2021 Jan 25;40:41. doi: 10.1186/s13046-021-01844-7 (PMC7830819; doi:10.1186/s13046-021-01844-7)

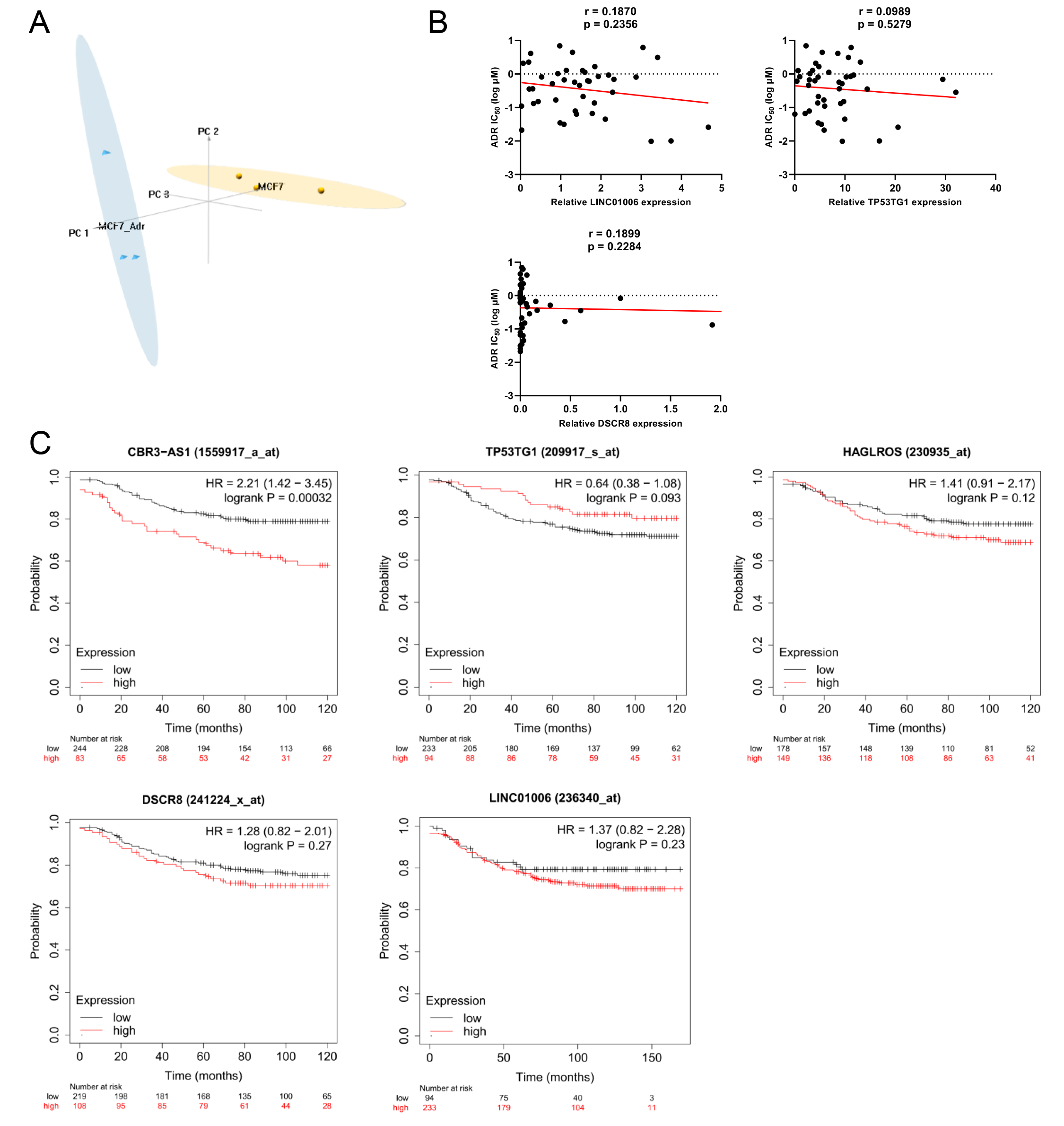

Supplement: Supplementary file 1 — Additional file 1: Figure S1. CBR3-AS1 is related to breast cancer drug resistance and poor prognosis. A PCA analysis of the microarray of MCF-7/ADR cells and MCF-7/ADR cells. B The correlation between the expression of lncRNAs and the drug resistance of breast cancer cells to ADR. C Kaplan–Meier analysis were performed on patients of the expression of 5 lncRNAs in GSE20685. [file 13046_2021_1844_MOESM1_ESM.tif]

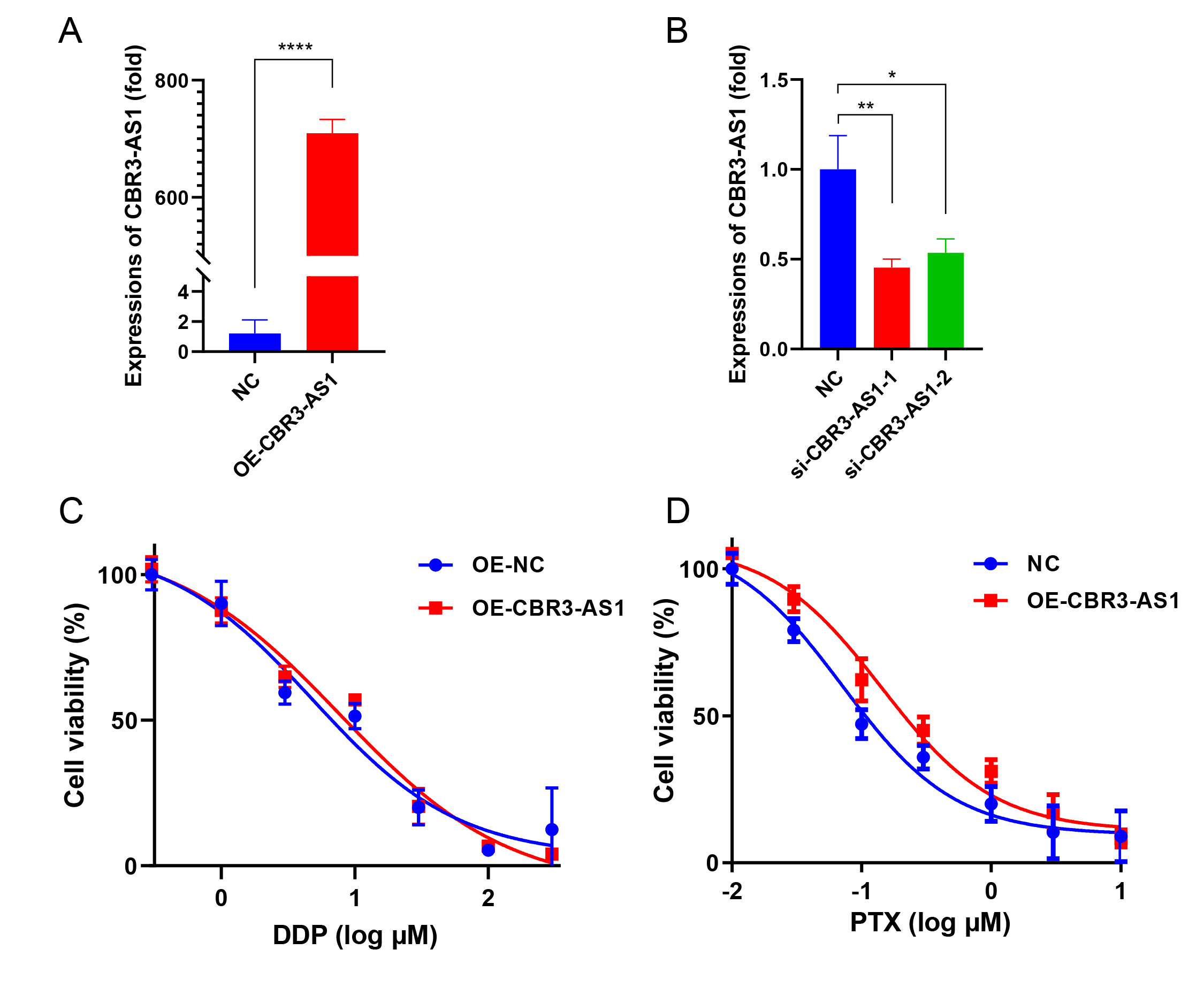

Supplement: Supplementary file 2 — Additional file 2: Figure S2. Construction of the cell models that interferes with the expression of CBR3-AS1. A After transfection of CBR3-AS1 plasmid, the expression of CBR3-AS1 in MCF-7 cells. B After transfection of CBR3-AS1 siRNAs, the expression of CBR3-AS1 in MCF-7/ADR cells. C The relative viability of the MCF-7 cells in cisplatin was detected by CCK-8 assays 48 h after transfection with Control and OE-CBR3-AS1. D The relative viability of the MCF-7 cells in paclitaxel was detected by CCK-8 assays 48 h after transfection with Control and OE-CBR3-AS1. *p < 0.05, **p < 0.01, ***p < 0.001, ****p < 0.0001. [file 13046_2021_1844_MOESM2_ESM.tif]

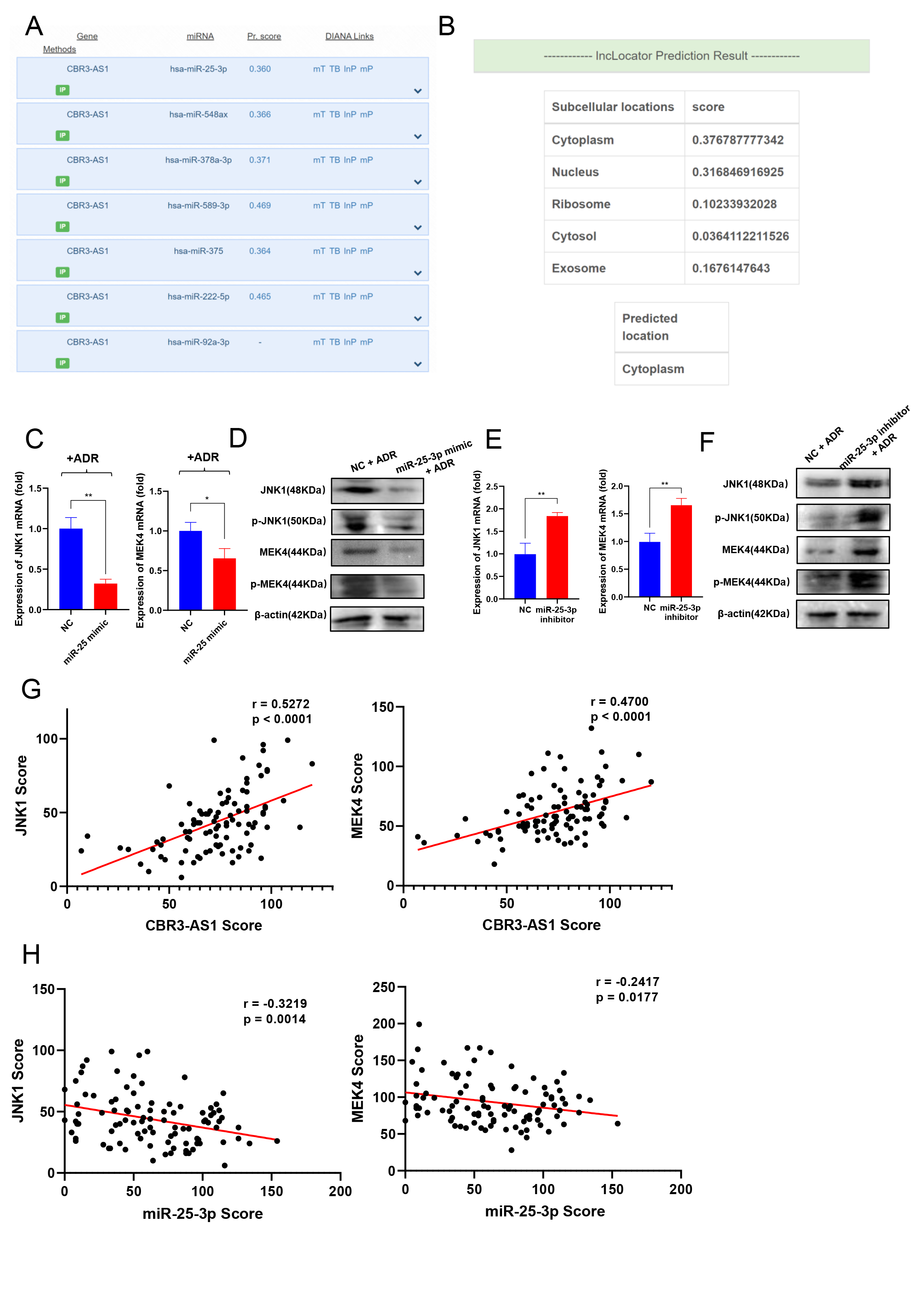

Supplement: Supplementary file 3 — Additional file 3: Figure S3. The relationship between CBR3-AS1, miR-25-3p and JNK1/MEK4. A Diana lncbase predicted the miRNAs combined with CBR3-AS1. B Subcellular localization of CBR3-AS1 predicted by the lnclocator website. C-D JNK1/MEK4 expression was measured by qRT-PCR after inhibited miR-25-3p in MCF-7 cells. E-F JNK1/MEK4 expression was measured by western blot and qRT-PCR after overexpressed miR-25-3p in MCF-7/ADR cells. G Linear correlation pattern showing a positive relationship between the expression of CBR3-AS1 and JNK1/MEK4. H Linear correlation pattern showing a negative relationship between the expression of miR-25-3p and JNK1/MEK4. *p < 0.05, **p < 0.01, ***p < 0.001, ****p < 0.0001. [file 13046_2021_1844_MOESM3_ESM.tif]
