# Supplementary material for: LncRNA CBR3-AS1 regulates of breast cancer drug sensitivity as a competing endogenous RNA through the JNK1/MEK4-mediated MAPK signal pathway
Source: J Exp Clin Cancer Res. 2021 Jan 25;40:41. doi: 10.1186/s13046-021-01844-7 (PMC7830819; doi:10.1186/s13046-021-01844-7)
Supplement: Supplementary file 4 — Additional file 4: Table S1. The sequences for primers used in the study. [file 13046_2021_1844_MOESM4_ESM.docx]

**Table S1. The sequences for primers used in the study.**

| β-action |
| --- |
| F:AGCCTCGCCTTTGCCGA |
| R:CTGGTGCCTGGGGCG |
| DSCR8 |
| F:AACATGGGTGGCAAAAAGAG |
| R:GTCACGGCATGAACTGAATG |
| CBR3-AS1 |
| F:AGTAGTTGCTTGTCCTAT |
| R:AAGTCAGTAAGTCCTAAGT |
| TP53TG1 |
| F:GCAGGAAGCGATGGTTAAGA |
| R:GGTGTAAGTGTTCGCCTGGT |
| HAGLROS |
| F:ACCTCTGAAGACAGGGCAAG |
| R:GCCTACTTCCTCCCACACAA |
| LINC01006 |
| F:GGTGAGGAGGATGGAATGAC |
| R:CGGTACAATGCCTGACACAC |
| JNK1 |
| F:TGTGTGGAATCAAGCACCTTC |
| R:AGGCGTCATCATAAAACTCGTTC |
| MEK4 |
| F:TCCCAATCCTACAGGAGTTCAA |
| R:CCAGTGTTGTTCAGGGGAGA |
| ABCB1 |
| F:AGGCCAACATACATGCCTTC |
| R:CCACCAGAGAGCTGAGTTCC |
| ABCG2 |
| F:TTCGGCTTGCAACAACTATG |
| R:TCCAGACACACCACGGATAA |
| ABCC1 |
| F:TGCCTGTTTTGGTAAAGAACTG |
| R:CTTGGAGGAGTACACAACCTTC |
