# Supplementary material for: LncRNA CBR3-AS1 regulates of breast cancer drug sensitivity as a competing endogenous RNA through the JNK1/MEK4-mediated MAPK signal pathway
Source: J Exp Clin Cancer Res. 2021 Jan 25;40:41. doi: 10.1186/s13046-021-01844-7 (PMC7830819; doi:10.1186/s13046-021-01844-7)
Supplement: Supplementary file 5 — Additional file 5: Table S2. Antibodies used for IHC and WB. [file 13046_2021_1844_MOESM5_ESM.docx]

**Table S2. Antibodies used for IHC and WB**

| **Antibody** | **Company/Provider** |
| --- | --- |
| anti-human ABCB1 | Abcam plc, Cambridge, UK |
| anti-human JNK1 | Proteintech Group,Inc.,IL,USA |
| anti-human MEK4 | Cell Signaling Technology, MA,USA |
| anti-human p-JNK1 | BOSTER Biological Technology co.ltd, UK |
| anti-human P-MEK4 | Bioss, Beijing, China |
| anti-human β-actin | Absin Bioscience Co.,Ltd, Shanghai, China |
| goat anti-mouse IgG | Invitrogen, CA , USA |
| goat anti-rabbit IgG | Invitrogen, CA , USA |
| goat anti-rabbit IgG-HRP | Earthox LLC., CA,USA |
| goat anti-mouse IgG-HRP | Earthox LLC., CA,USA |
